# Probiotic Yeasts Inhibit Virulence of Non*-albicans Candida* Species

**DOI:** 10.1128/mBio.02307-19

**Published:** 2019-10-15

**Authors:** Lohith Kunyeit, Nawneet K. Kurrey, K. A. Anu-Appaiah, Reeta P. Rao

**Affiliations:** a Department of Microbiology and Fermentation Technology, CSIR—Central Food Technological Research Institute (CFTRI), Mysore, India; b Academy of Scientific and Innovative Research (AcSIR), CFTRI, Mysore, India; c Department of Biology and Biotechnology, Worcester Polytechnic Institute, Worcester, Massachusetts, USA; d Department of Biochemistry, CSIR—Central Food Technological Research Institute (CFTRI), Mysore, India; Duke University

**Keywords:** probiotic yeasts, plastic adhesion assay, Caco-2 cell monolayer, mixed-species *Candida* biofilm, *Candida tropicalis*, *Candida krusei*, *Candida glabrata*, *Candida parapsilosis*, *Candida auris*, *Caenorhabditis elegans*, *Candida albicans*, biofilm

## Abstract

Systemic infections of *Candida* species pose a significant threat to public health. Toxicity associated with current therapies and emergence of resistant strains present major therapeutic challenges. Here, we report exploitation of the probiotic properties of two novel, food-derived yeasts, Saccharomyces cerevisiae (strain KTP) and Issatchenkia occidentalis (strain ApC), as an alternative approach to combat widespread opportunistic fungal infections. Both yeasts inhibit virulence traits such as adhesion, filamentation, and biofilm formation of several non-*albicans Candida* species, including Candida tropicalis, Candida krusei, Candida glabrata, and Candida parapsilosis as well as the recently identified multidrug-resistant species Candida auris. They inhibit adhesion to abiotic surfaces as well as cultured colon epithelial cells. Furthermore, probiotic treatment blocks the formation of biofilms of individual non-*albicans Candida* strains as well as mixed-culture biofilms of each non-*albicans Candida* strain in combination with Candida albicans. The probiotic yeasts attenuated non-*albicans Candida* infections in a live animal. *In vivo* studies using Caenorhabditis elegans suggest that exposure to probiotic yeasts protects nematodes from infection with non-*albicans Candida* strains compared to worms that were not exposed to the probiotic yeasts. Furthermore, application of probiotic yeasts postinfection with non-*albicans Candida* alleviated pathogenic colonization of the nematode gut. The probiotic properties of these novel yeasts are better than or comparable to those of the commercially available probiotic yeast Saccharomyces boulardii, which was used as a reference strain throughout this study. These results indicate that yeasts derived from food sources could serve as an effective alternative to antifungal therapy against emerging pathogenic *Candida* species.

## Abstract (precis)

Non-*albicans Candida*-associated infections have emerged as a major risk factor in the hospitalized and immunecompromised patients. Besides, antifungal-associated complications occur more frequently with these non-*albicans Candida* species than with C. albicans. Therefore, as an alternative approach to combat these widespread non-*albicans Candida*-associated infections, here we showed the probiotic effect of two yeasts, Saccharomyces cerevisiae (strain KTP) and Issatchenkia occidentalis (ApC), in preventing adhesion and biofilm formation of five non-*albicans Candida* strains, Candida tropicalis, Candida krusei, Candida glabrata, Candida parapsilosis, and Candida auris. The result would influence the current trend of the conversion of conventional antimicrobial therapy into beneficial probiotic microbe-associated antimicrobial treatment.

## INTRODUCTION

Opportunistic invasive *Candida* infections present a major public health threat, especially in immunocompromised populations or healthy individuals with implanted medical devices (1). Impairment of immune functions permits the pathogen to penetrate the submucosal tissue of gastrointestinal tract and disseminate to the internal organs, resulting in life-threatening systemic infections (2). While the most prominent etiological agent is Candida albicans, other yeasts of the genus *Candida*, collectively referred to as non-*albicans Candida* (NAC), have also been associated with nosocomial infections (3). For example, C. tropicalis, C. parapsilosis, and C. glabrata are associated with 35% to 65% of all systemic *Candida* infections (4), while C. krusei is fluconazole resistant (5). Infection with any non-*albicans Candida* strain increases patient morbidity, and coinfections with C. tropicalis and C. glabrata have high (40% to 70%) mortality rates (4).

More recently, evolution of antifungal-resistant strains of C. albicans as well as non-*albicans Candida* has emerged as a critical issue (3, 6, 7). The global emergence of Candida auris as a multidrug-resistant fungal pathogen with high mortality rates (8) has prompted national and international surveillance programs. The antifungal agents used to treat *Candida* infections have a myriad of deleterious side effects due to their similarity to eukaryotic host cells. This has led to a growing recognition that alternative therapy for opportunistic pathogens is desirable. Food-derived probiotic yeasts present a safe and cost-effective method to keep *Candida* in check with improved health and wellness for the patient.

Microbes often exist in communities where cells attach to abiotic surfaces or host cells. The adherent cells subsequently become embedded within an extracellular matrix to form a complex ecosystem called a biofilm. Surface adhesion is usually the first step of an infection, while biofilms form a physical barrier against the drugs, directly contributing to the antifungal resistance (9–11). *Candida* biofilms on abiotic materials such as medical devices—urinary and central venous catheters, pacemakers, mechanical heart valves, joint prostheses, contact lenses—have been shown to contribute to deadly infections (12–15). Furthermore, *Candida* biofilms have been shown to damage epithelial surfaces, causing vaginitis or thrush, and in rare cases may breach the vascular endothelium and progress to endocarditis (16, 17). Therefore, methods designed to restrict adhesion and, ultimately, biofilm formation are effective therapies.

Found in many fermented foods and beverages, yeasts are an inevitable part of the daily diet of humans in most cultures. Yeast-based probiotics are especially desirable because they are naturally resistant to most antibiotics, which allows them to persist in the gastrointestinal (GI) tract during an antibiotic regimen when the bacterial microflora may be compromised. The use of Saccharomyces cerevisiae is particularly attractive for probiotic applications and is generally regarded as safe (GRAS) by the U.S. Food and Drug Administration (FDA) (18). Recently published preclinical and clinical studies support the use of probiotics against C. albicans (19), and Saccharomyces boulardii is already commercially available. A probiotic cocktail of yeast and bacteria in combination with prebiotics was found to reduce colonization of C. albicans in preteen children (20). Additionally, recent evidence suggests that vaginal administration of S. cerevisiae in mice significantly reduced C. albicans colonization during vulvovaginal candidiasis (VVC) (21). However, there is limited knowledge about the effects of probiotic yeasts on non-*albicans Candida* strains. Here, we tested the effects of two novel probiotic yeasts, Saccharomyces cerevisiae (strain KTP) and Issatchenkia occidentalis (strain ApC), that were derived from food sources (22). Here, we used multiple readouts, including *in vitro* and *ex vivo* assays and analyses of morphological transition and biofilm formation of four non-*albicans Candida* strains, C. tropicalis, C. krusei, C. glabrata, and C. parapsilosis, to demonstrate the efficacy of probiotic yeast application. We also used Caenorhabditis elegans as a whole-animal infection model to study the effects of exposure to probiotic yeasts. Finally, we demonstrate that the use of probiotic yeasts represents an effective method to control the multidrug-resistant species Candida auris.

## RESULTS

We have previously reported the isolation and characterization of two probiotic yeasts, S. cerevisiae (strain KTP; accession no. MH142729) and I. occidentalis (strain ApC; accession no. KF551991) from toddy and apple cider, respectively (22). Here, we tested the probiotic potential of these yeasts against non-*albicans Candida* species C. tropicalis, C. glabrata, C. krusei, and C. parapsilosis as well as the recently identified multidrug-resistant species C. auris. To fully appreciate the probiotic effects of the yeasts S. cerevisiae and I. occidentalis on C. tropicalis, C. glabrata, C. krusei, and C. parapsilosis, the assays reported in this study were typically performed under three different sets of conditions: preinoculation, where treatment with probiotic yeasts was performed prior to application of the *Candida* strains; coinoculation, where probiotic yeasts and *Candida* strains were simultaneously applied; and postinoculation, where probiotic yeast treatment was performed after the application of *Candida* strains (treatment 60 and 90 min after adhesion and biofilm initiation respectively).

### S. cerevisiae and I. occidentalis inhibit adhesion of non-*albicans Candida* species.

**FIG 1:**
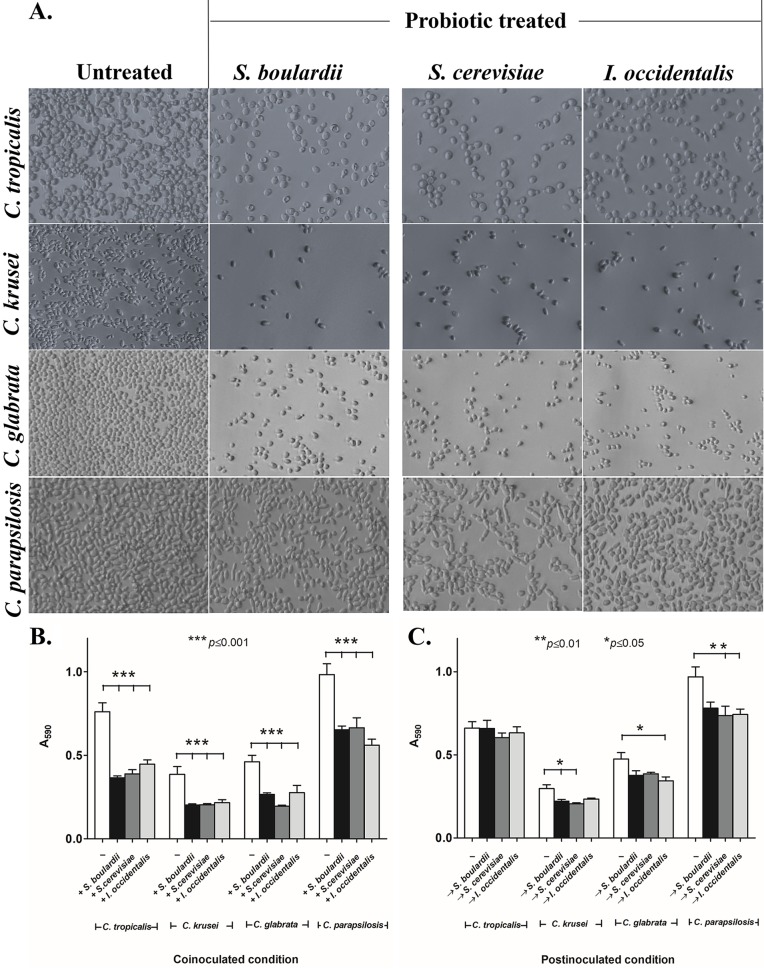
Probiotic treatment reduces adhesion of non-*albicans Candida* strains on abiotic surfaces. (A) Images show effects of potential probiotic yeasts S. cerevisiae and I. occidentalis and reference strain S. boulardii on adhesion of C. tropicalis, C. krusei, C. glabrata, and C. parapsilosis under coinoculation conditions. (B and C) Adhesion of non-*albicans Candida* strains to plastic surfaces was reduced in the presence of probiotic yeasts under coinoculation conditions (B) and postinoculation conditions (C). For the coinoculation conditions (indicated with a plus sign), probiotic yeasts and non-*albicans Candida* strains were incubated together for 3 h. For the postinoculation conditions (indicated with a vertical arrow), non-*albicans Candida* strains were applied to an abiotic surface for 60 min prior to seeding of probiotic isolates and were incubated for an additional 120 min. Crystal violet (0.5%) staining was used to quantify adhered non-*albicans Candida* cells on abiotic surfaces.

Previous studies revealed that probiotic dosage is a crucial consideration for treatment (23). Therefore, we empirically determined that 10^8^ cells/ml was the effective dosage for S. cerevisiae or I. occidentalis. This effective dosage is in line with that observed for the commercially available probiotic yeast S. boulardii, the reference strain used in our studies. Plastic surfaces that were pretreated with 10^8^ cells/ml of either of the probiotic yeast species inhibited adhesion of the non-*albicans Candida* strains by 80% to 90% compared to untreated surfaces (see Fig. S1 in the supplemental material). We and others have confirmed that inhibition of adhesion decreases in a dose-dependent manner when probiotics are applied at concentrations lower than the effective dose. When probiotic yeasts were coincubated with the *Candida* strains, adhesion of C. krusei, C. glabrata, and C. parapsilosis was reduced by 43% to 52% (*P* < 0.05) and that of C. tropicalis by 33% to 42% (*P* < 0.05) compared to the controls that were not coincubated with probiotics (Fig. 1A and B). The extent of inhibition seen with the novel probiotic yeasts was similar to the level seen with after treatment with the commercially available reference probiotic yeast S. boulardii, which exhibited a 43% to 53% reduction in the adhesion of C. tropicalis, C. krusei, and C. glabrata and a 34% reduction of adhesion of C. parapsilosis compared with untreated controls under identical conditions. To test whether metabolic activity was necessary for probiotic function, we exposed heat-killed probiotic yeasts to non-*albicans Candida* strains. Our results indicated that inactivation of probiotic yeasts renders them inactive, allowing the non-*albicans Candida* strains to adhere to abiotic surfaces (Fig. S2). Together, these results suggest that metabolically active probiotic yeasts are effective prophylactics. To study the effect of probiotic yeasts as a treatment, we tested whether the application of probiotic yeasts would be able to displace *Candida* strains that had already attached to the plastic surface of 96-well plates (postinoculation condition). Our results indicate that the probiotic yeasts were unable to displace C. tropicalis and displaced C. krusei, C. glabrata, and C. parapsilosis only minimally (by 5% to 25%, *P* < 0.05) compared to an untreated control population (Fig. 1C). Together, our results indicate that the novel probiotic yeasts S. cerevisiae and I. occidentalis are able to inhibit attachment of a variety of *Candida* strains, thereby preventing formation of biofilms. These novel probiotic yeasts are as effective as the commercially available probiotic yeast S. boulardii. These probiotics are, however, not effective once *Candida* has attached and formation of biofilms has been initiated.

10.1128/mBio.02307-19.1 FIG S1 Preinoculation of probiotic yeasts inhibits adhesion of non-*albicans Candida* to abiotic surfaces. Download FIG S1, TIF file, 1.0 MB. Copyright © 2019 Kunyeit et al. 2019 Kunyeit et al. This content is distributed under the terms of the Creative Commons Attribution 4.0 International license.

10.1128/mBio.02307-19.2 FIG S2 Effect of heat-killed probiotic yeasts S. boulardii, S. cerevisiae, and I. occidentalis on adhesion of non-*albicans Candida* yeasts. Download FIG S2, TIF file, 2.3 MB. Copyright © 2019 Kunyeit et al. 2019 Kunyeit et al. This content is distributed under the terms of the Creative Commons Attribution 4.0 International license.

### S. cerevisiae and I. occidentalis inhibit biofilm formation of non-*albicans Candida* species.

**FIG 2:**
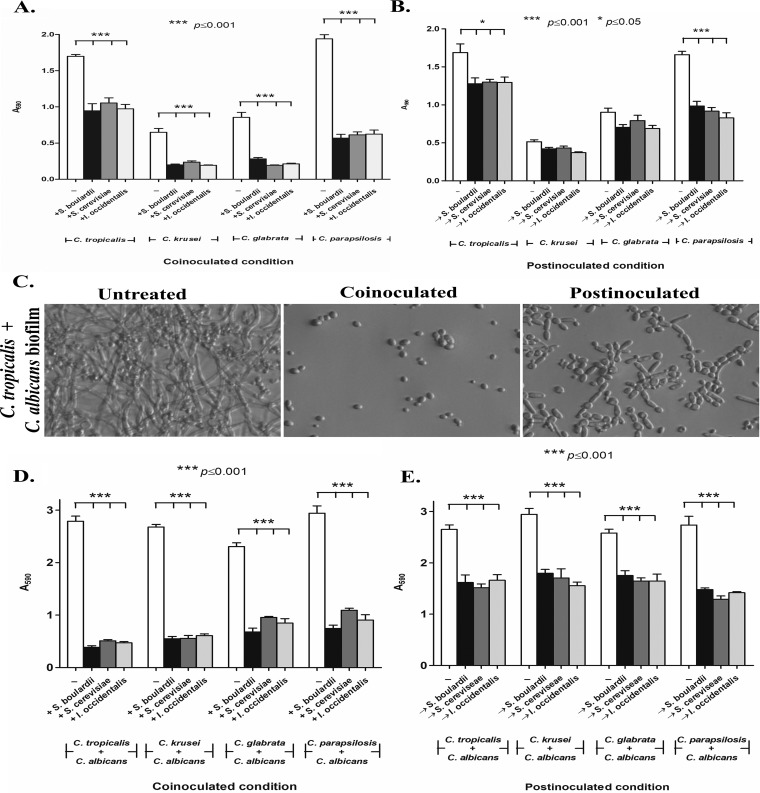
Probiotic yeasts S. cerevisiae, I. occidentalis, and S. boulardii prevented biofilm formation of non-*albicans Candida* species in both monoculture and mixed-culture biofilms with C. albicans. (A) Coinoculation of probiotics with non-*albicans Candida* strains for 24 h at 37°C. (B) For postinoculation of probiotics, non-*albicans Candida* strains were incubated for 90 min, and nonadherent cells were removed and subsequently treated with probiotic yeasts for 24 h at 37°C. Crystal violet (0.5%) staining was used to quantify the biofilm. (C) Images show the inhibitory effect of coinoculated probiotic treatment and postinoculation treatment (treatment 90 min after biofilm initiation). (D and E) Probiotic treatment inhibited mixed-culture biofilms of non-*albicans Candida* with C. albicans under conditions of coinoculation (D) or 90 min postinoculation, when biofilm formation had been initiated (E).

Next, we wanted to test the effect of S. cerevisiae and I. occidentalis on biofilm formation in three stages of biofilm development, defined as follows: early biofilms formed after 90 min; intermediate biofilms formed after 24 h; and mature biofilms formed after 48 h. The putative probiotic yeasts S. cerevisiae and I. occidentalis (10^8^ cells/ml) were applied to preformed biofilms consisting of each of the non-*albicans Candida* strains. Results were compared to those seen with untreated controls as well as treatment with the commercially available, reference probiotic strain S. boulardii. For all non-*albicans Candida* biofilms tested, probiotic yeasts inhibited further development of early biofilms compared to the intermediate and mature stages of biofilms (Fig. 2A and B). In addition, probiotic strains coincubated with C. krusei, C. glabrata, and C. parapsilosis exhibited 65% to 70% inhibition of biofilm formation whereas C. tropicalis biofilms were inhibited by 44%.

Biofilms, in nature, are found as surface-attached communities of microorganisms. Therefore, we tested the ability of the probiotic yeasts to inhibit biofilms formed by a mixed culture consisting of each of the non-*albicans Candida* strains in combination with C. albicans. Early (90-min) stages of the mixed-species biofilms showed significant (48% to 81%) reductions upon treatment with putative probiotic yeasts (Fig. 2D and E). However, intermediate (24-h) and mature (48-h) biofilms were not affected by probiotic treatment even when the duration of treatment was increased (Fig. S3). Furthermore, exposure to probiotics decreased the overall metabolic activity in the biofilm even though the biomass was not affected (Fig. S3C and D). Together, these results suggest that the putative probiotic yeasts are able to inhibit non-*albicans Candida* biofilms when applied early in the development.

10.1128/mBio.02307-19.3 FIG S3 Probiotic yeasts do not alter the course of biofilm formation when applied on mature biofilms (A and B) but instead decrease the metabolic activity of the biofilms (C and D). Mature, 24-h-old biofilms of monoculture non-*albicans Candida* strains or mixed culture of non-*albicans Candida* and C. albicans were treated with probiotic yeasts S. boulardii, S. cerevisiae, and I. occidentalis and incubated for an additional 24 h. The relative biomasses of monoculture non-*albicans Candida* biofilms (A) and of cultures of non-*albicans Candida* mixed with C. albicans (B) were assessed using crystal violet staining. Levels of metabolic activity of monoculture non-*albicans Candida* biofilms (C) and mixed culture of non-*albicans Candida* with C. albicans (D) were measured using MTT assay. Download FIG S3, TIF file, 3.4 MB. Copyright © 2019 Kunyeit et al. 2019 Kunyeit et al. This content is distributed under the terms of the Creative Commons Attribution 4.0 International license.

### Putative probiotics reduced filamentation of non-*albicans Candida* strains.

**FIG 3:**
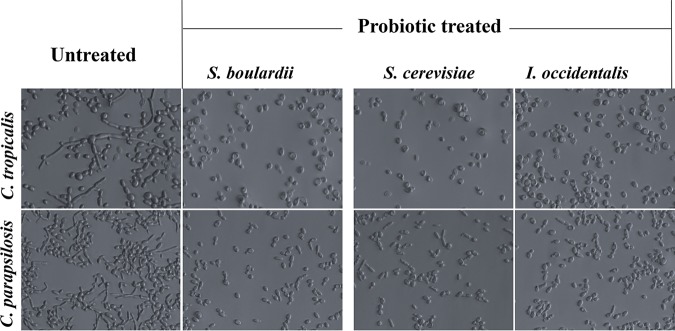
Probiotic yeasts S. cerevisiae, I. occidentalis, and S. boulardii inhibited morphological transition of C. tropicalis (upper row) and C. parapsilosis (bottom row). Probiotic yeasts and non-*albicans Candida* were coincubated for 24 h, following which unattached cells were removed and photographed using a bright-field microscope.

Filamentation is a key virulence factor for various fungi of the *Candida* species and is positively correlated with adhesion and biofilm formation. Therefore, we wanted to test the effect of the putative probiotics S. cerevisiae and I. occidentalis on cell morphology and filamentation. Levels of hyphal development of C. tropicalis and C. parapsilosis were significantly inhibited upon treatment with probiotic yeasts at 10^8^/ml (Fig. 3). C. krusei and C. glabrata were not tested since they do not exhibit morphological transition. These results bolster the hypothesis that food-derived yeasts function as effective probiotics against pathogenic species of *Candida*.

### S. cerevisiae and I. occidentalis inhibited adhesion of non-*albicans Candida* strains to cultured epithelial cells from human colon.

It is normal to find *Candida* in small amounts in the mouth, intestines, and skin. Overgrowth of *Candida*, however, can be problematic. In the context of the human gastrointestinal tract, *Candida* cells first attach to the epithelial cells and then invade deeper tissues. To test the ability of the putative probiotic yeasts to prevent attachment of *Candida* to human epithelial cells, we performed cell adhesion assays using monolayers of Caco-2 epithelial cells derived from human colon. The following three conditions were tested: the preinoculation condition, where Caco-2 cells were exposed to probiotics prior to exposure to non-*albicans Candida* strains; the coinoculation condition, where Caco-2 cells were exposed to non-*albicans Candida* and probiotic yeasts simultaneously; and the postinoculation condition, where Caco-2 cells were exposed to non-*albicans Candida* strains and then treated with probiotic yeasts. Our results indicated that adhesion to Caco-2 monolayer was inhibited by 95% to 99% under the preinoculation condition (data not shown). Under the coinoculation and postinoculation conditions, 72% to 98% of non-*albicans Candida* strains were inhibited (*P* < 0.05) (Table 1). Interestingly, C. glabrata and C. parapsilosis showed poor adhesion to Caco-2 monolayer compared to C. tropicalis and C. krusei. These results suggest that the probiotic yeasts prevent the attachment of non-*albicans Candida* yeasts to cultured human epithelial cells.

**TABLE 1:** Treatment with probiotic strains reduced adhesion of non-*albicans Candida* strains to Caco-2 cell monolayer^*a*^

NAC species	Number of adherent probiotic cells (CFU/ml)							
	Coinoculation condition				Postinoculation condition			
	—	+S. boulardii	+S. cerevisiae	+I. occidentalis	—	→S. boulardii	→S. cerevisiae	→I. occidentalis
C. tropicalis	19,118 ± 596	401 ± 82	411 ± 85	390 ± 87	21,335 ± 314	826 ± 46	445 ± 18	498 ± 11
C. krusei	20,680 ± 980	550 ± 98	441 ± 82	346 ± 68	15,255 ± 731	948 ± 83	493 ± 22	1,000 ± 54
C. parapsilosis	1,307 ± 203	184 ± 40	186 ± 37	205 ± 17	5,504 ± 94	439 ± 45	462 ± 37	480 ± 32
C. glabrata	1,757 ± 168	257 ± 81	261 ± 28	171 ± 41	832 ± 33	374 ± 21	386 ± 11	424 ± 21

a For the coinoculation condition (+), probiotic yeasts and non-*albicans Candida* strains were coinoculated with monolayers of Caco-2 cells and incubated for 3 h. For the postinoculation condition (→), non-*albicans Candida* strains were applied on an epithelial layer of Caco-2 cells for 60 min prior to inoculation of probiotic yeasts and incubated for an additional 120 min. Candida chrome agar was used to assess CFU of adhered non-*albicans Candida* on an epithelial layer of Caco-2 cells. All values are expressed as means ± SD. All the values represent statistical significance at *P* values of <0.05 in comparison to the results determined for the untreated control group (—).

### Application of probiotic yeasts protects C. elegans from non-*albicans Candida* species.

To further investigate the protective phenotypes of the probiotic yeasts in a live animal, we used C. elegans as a model host. C. elegans mimics key aspects of human intestinal physiology, including the presence of polarized microvillus-containing cells (24). The life span of C. elegans reared on a diet of probiotic yeasts (S. cerevisiae, I. occidentalis, or S. boulardii) was similar to that seen with those fed the standard diet of Escherichia coli OP50 (Fig. S4), suggesting that the probiotic treatment is benign and does not affect normal development of the worm.

10.1128/mBio.02307-19.4 FIG S4 Probiotic effect of yeasts S. boulardii, S. cerevisiae, and I. occidentalis on C. elegans survival. Download FIG S4, TIF file, 2.0 MB. Copyright © 2019 Kunyeit et al. 2019 Kunyeit et al. This content is distributed under the terms of the Creative Commons Attribution 4.0 International license.

**FIG 4:**
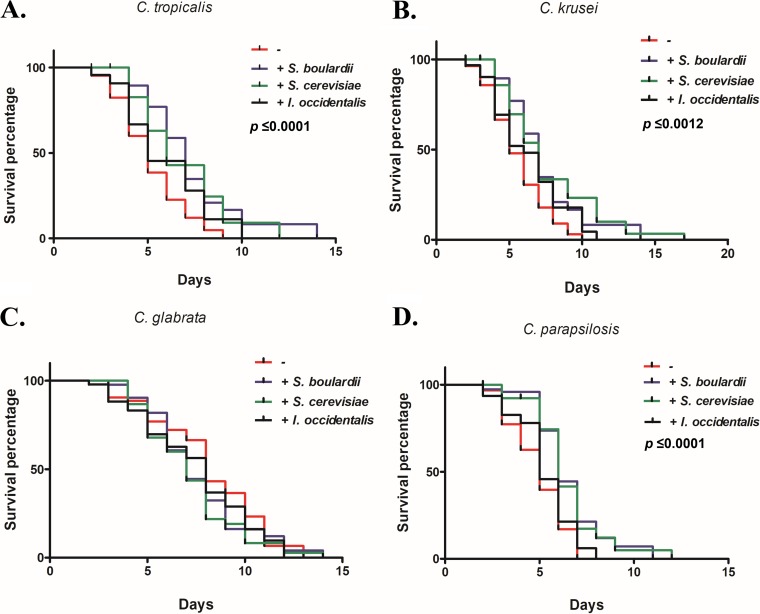
Simultaneous exposure of probiotic yeasts S. boulardii, S. cerevisiae, and I. occidentalis increased the life span of C. tropicalis (A), C. krusei (B), and C. parapsilosis (D) but not C. glabrata (C). C. elegans worms were fed a mixture of probiotic yeasts and non-*albicans Candida.* Live and dead worms were scored for survival each day and compared to worms fed a diet consisting only of non-*albicans Candida* (untreated group).

Coinfection of C. elegans with probiotic yeast along with C. tropicalis, C. krusei, or C. parapsilosis exhibited a life span that was extended (by 5 to 6 days) compared to the life span seen with untreated control worms (Fig. 4). Furthermore, CFU levels of non-*albicans Candida* strains recovered from the gut of probiotic-treated worms were significantly decreased compared to the levels seen with the untreated group (Fig. S5). Together, these results suggest that probiotic treatment inhibits the gut colonization of non-*albicans Candida* strains and extends the nematode life span. In addition, we tested conditions under which the probiotic yeasts were administered after C. elegans worms were infected with C. tropicalis, C. krusei, or C. parapsilosis. The nematodes that were treated with probiotic yeasts postinfection with non-*albicans Candida* were able to reduce colonization, with no CFU recovered on day 5 after treatment with probiotics. We also noted that worms treated with yeasts of the genus *Saccharomyces* (both S. cerevisiae and S. boulardii) resisted pathogenic insult better than those treated with the non-*Saccharomyces* yeast I. occidentalis.

10.1128/mBio.02307-19.5 FIG S5 Probiotic treatment reduces colonization of the nematode gut with non-*albicans Candida* species. Download FIG S5, TIF file, 3.1 MB. Copyright © 2019 Kunyeit et al. 2019 Kunyeit et al. This content is distributed under the terms of the Creative Commons Attribution 4.0 International license.

### Effect of application of probiotic yeasts on virulence of C. auris.

**FIG 5:**
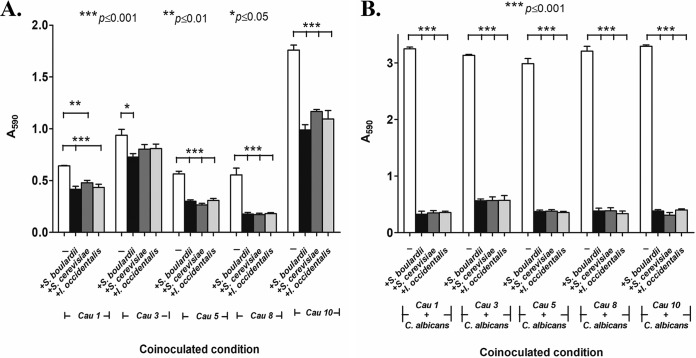
Effect of probiotics treatment on adhesion (A) and biofilm formation (B) of C. auris. Probiotic yeasts S. cerevisiae and I. occidentalis as well as reference strain S. boulardii and C. auris were coinoculated for 3 h and 24 h at 37°C. Adhesion and biofilm formation were quantified by crystal violet (0.5%) staining after unattached cells were removed by washing. (A) Coincubation of probiotic yeasts decreased adhesion of C. auris to abiotic surfaces. (B) Coinoculation of probiotic yeasts inhibited mixed-culture biofilms of C. auris with C. albicans.

Recently, Candida auris has emerged as a multidrug-resistant superbug that presents a serious global health threat, especially among people with a weakened immune system (25). It attaches to abiotic surfaces, prompting hospitals to take extraordinary decontamination measures, including removal of ceiling and floor tiles, to eradicate it. It has been suggested that the recent rise in C. auris infections is in part due to the overuse of antimicrobial agents that wipe out competing microbes, giving drug-resistant C. auris a chance to overgrow. Therefore, we tested the ability of competing probiotic yeasts to inhibit adhesion of C. auris (obtained from the U.S. Centers for Disease Control). We tested C. auris representing each of the clades. Coinoculation of C. auris strains with probiotics resulted in inhibition of adhesion by 44% to 62% (Fig. 5A), while treatment after probiotics after C. auris had attached to the surface (postinoculation condition) showed a modest decrease in adhesion (34% to 40%; data not shown). We also tested the effect of probiotic treatment on mixed-species biofilms of C. auris and C. albicans, since monocultures of C. auris do not form rich biofilms. Our results revealed that coinoculation of S. cerevisiae or I. occidentalis inhibited mixed-species biofilms of C. auris and C. albicans by 90% (Fig. 5B) and by 27% to 46% (data not shown) under the postinoculation condition. Together, these results indicate that probiotic yeasts inhibit adhesion and biofilm formation of C. auris.

### The secretome of probiotic yeasts inhibited adhesion of non-*albicans Candida* to abiotic surfaces.

**FIG 6:**
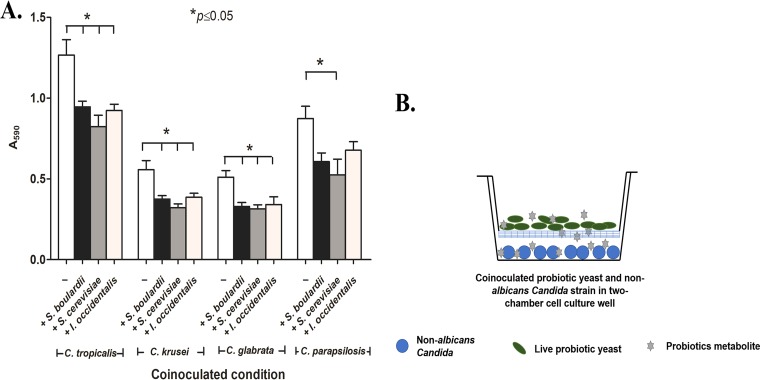
(A) The effect of cell-free probiotic metabolites on adhesion of non-*albicans Candida* strains was quantified using crystal violet staining. (B) Graphic representation of experimental setup where probiotic yeast were inoculated into the upper chamber of the cell insertion section and non-*albicans Candida* strains were maintained in the lower chamber; probiotic soluble metabolites freely diffused through a 0.4-μl-pore-size membrane into and all over the media, including the lower compartment where the non-*albicans Candida* cells were inoculated.

To probe the mechanism of probiotic action, we tested whether the secretome of the probiotic yeasts retained the ability to inhibit adhesion of non-*albicans Candida* to abiotic surfaces. A two-chamber cell culture insert was used where probiotic yeasts were maintained in the upper chamber and were separated from the lower chamber by a 0.4-μm-pore-size membrane that allowed diffusion of small bioactive molecules to the lower chamber containing non-*albicans Candida* cells (Fig. 6). Our results indicate that a soluble metabolite(s) present in the secretome of probiotic yeasts was able to partially inhibit adhesion of non-*albicans Candida* (by 22% to 30%) compared to the untreated control. In addition, spent media buffered to neutral pH (pH 7) retained the adhesion-inhibitory effect (18% to 31%), suggesting that antiadhesion nature of probiotic yeast is likely due to a bioactive metabolite(s) and not to the acidic nature of the spent media. These results suggest that a secreted metabolite(s) of probiotic yeasts is able to inhibit the virulence of non-*albicans Candida*.

## DISCUSSION

Biofilm-related clinical complications are a major issue in the health sector. Naturally occurring biofilms are usually polymicrobial, where interaction between microbes may be synergistic or antagonistic. We and others have observed that non-*albicans Candida* species such as Candida parapsilosis, C. pseudotropicalis, and C. glabrata produced immature biofilms when grown as monocultures compared to C. albicans (26). Fungi of the *Candida* species have been shown to synergize with bacteria in the oral cavity for adhesion and biofilm formation (27). Likewise, C. glabrata relies on C. albicans for initial adhesion and biofilm development (28). On the other hand, *Lactobacillus* species have been shown to inhibit the initial stages of biofilm in mixed cultures (29). We used several *in vitro* and *ex vivo* tools, including analyses of adhesion to abiotic surfaces and cultured epithelial cells derived from human colon (Caco-2 cell), morphological transition, and biofilm formation, to assess effects of our potential probiotic strains against non-*albicans Candida* stains. Our results converge on the notion that probiotic yeasts inhibit adhesion and decrease metabolic activity of biofilms, thereby controlling growth and colonization of pathogenic *Candida*.

We used the nematode Caenorhabditis elegans as a live host model (30) since facets of its innate immune system are faithfully conserved in humans (31). We demonstrated that nematodes treated with probiotic yeasts are better able to withstand pathogenic insult from several non-*albicans Candida* species. Similarly, Lactobacillus acidophilus was previously shown to significantly decrease the colonization of infectious Gram-positive bacteria in C. elegans gut and to enhance the life span of the worm (32).

Probiotic action can be attributed to physical and/or chemical factors. Here, we provide evidence of the chemical nature of probiotic action since cell-free secretomes of probiotic yeasts retained inhibitory activity. We propose that a secondary metabolite(s) produced by the probiotic yeasts is secreted into the milieu, where it interferes with the pathogenic program of the non-*albicans Candida* species. Other reports have demonstrated that short-chain fatty acids or bacteriocins showed significant anti-*Candida* activity for various *Candida* species (19, 33–35). Probiotics may also pose a physical barrier by binding surface proteins that promote pathogen attachment or compete for limited nutrients. We showed that metabolically inactive probiotic yeasts retained minimal inhibitory effect, suggesting that the live probiotic cells and their metabolites likely act synergistically. Prior studies using various *in vitro* and *in vivo* models have also revealed synergistic mechanisms that involve immune simulation and competitive binding (21, 36–38).

Small molecules (such as filastatin, farnesoic acid, and gymnemic acids) and hydrophilic or antibody-coated compounds have been proposed as biotherapeutic agents but are associated with significant safety concerns (39–42). Therefore, probiotics such as S. cerevisiae and I. occidentalis have the potential to inhibit key virulence traits of the most common non-*albicans Candida* species, i.e., C. tropicalis, C. krusei, C. glabrata, and C. parapsilosis. To meet the growing need for treatment options for biofilm-associated clinical complications, these food-derived yeasts represent a safe and attractive alternative to conventional treatment for *Candida* infections.

## MATERIALS AND METHODS

### Chemicals, yeast strains, and growth conditions.

Standard yeast culture conditions were performed as described previously by Guthrie and Fink (43). Medium and/or medium components RPMI 1640, Dulbecco’s modified Eagle medium (DMEM), and fetal bovine serum (FBS) and chemicals such as MTT [3-(4,5-dimethyl-2-thiazolyl)-2,5-diphenyl-2H-tetrazolium bromide] were obtained from Sigma-Aldrich.

Saccharomyces cerevisiae (strain KTP; accession number MH142729) and Issatchenkia occidentalis (ApC; accession number KF551991) were isolated from toddy and fermented apple juice, respectively. The commercially available Saccharomyces cerevisiae var. *boulardii* (NCDC363) strain obtained from National Collection Centre for Dairy Cultures, India, was used as a reference strain in the study. The non-*albicans Candida* species C. tropicalis (MYA 3404), Candida krusei (44), C. glabrata (44), and C. parapsilosis (CDC317) were used in the study. The C. albicans strain (SC5314) was used for mixed-culture biofilm studies with non-*albicans Candida* stains. All strains were cultured in yeast extract-peptone-dextrose (YPD) media at 30°C for 24 h. RPMI 1640 containing l-glutamine, phenol red, 0.2% glucose, and 0.165 M MOPS (morpholinepropanesulfonic acid) buffer without sodium bicarbonate was used to test for plastic adhesion and biofilm formation.

### Assays to monitor adhesion of non-*albicans Candida* species. (i) *In vitro* plastic adhesion assay and biofilm formation assay.

In order to investigate probiotic effects on non-*albicans Candida* adhesion, we used a published protocol (45, 46) with minor modifications. Briefly, 10^7^ cells/ml of any *Candida* strain was treated with 10^8^ cells/ml of S. cerevisiae, I. occidentalis, or the reference strain of S. boulardii. In order to understand the limitations and effects of the probiotic treatment against non-*albicans Candida* strain, the assays were performed under preinoculation, coinoculation, and postinoculation conditions. First, a preexposure condition where probiotic yeast were inoculated into 96-well plates for 60 min (representing a doubling time of approximately 1) was applied. After 60 min, non-*albicans Candida* strains were introduced and incubated for an additional 120 min at 37°C with mild shaking (90 rpm). Second, the three probiotic strains S. boulardii, S. cerevisiae, and I. occidentalis were coinoculated with non-*albicans Candida* strains and incubated for 3 h. Third, a postinoculation condition where the non-*albicans Candida* strains were inoculated in the 96-well microtiter plates for 60 min was applied. Then, test probiotic isolates S. cerevisiae and I. occidentalis or cells of the reference strain S. boulardii were seeded on the adhered non-*albicans Candida* cells and incubated further 120 min under the conditions mentioned above. After treatment, plates were washed three times with phosphate-buffered saline (PBS; pH 7.4) to remove nonadherent cells. Finally, plates were air-dried and incubated with 50 μl of 0.5% crystal violet for 45 min, followed by washing with PBS to remove the excess crystal violet. The stained cells were destained with 95% (vol/vol) ethanol, and absorbance was measured at 590 nm to analyze the number of adhered cells.

**(ii) *In vitro* biofilm formation assay.** The effect of probiotic yeasts was tested on biofilms initiated as monocultures of non-*albicans Candida* species or as a mixed-culture biofilm with C. albicans. Furthermore, biofilms were tested at the early, intermediate, and mature stages of development. Non-*albicans Candida* strains were coincubated with yeast (S. cerevisiae, I. occidentalis, or reference strain S. boulardii) for 24 h at 37°C. Under the postinoculation condition, non-*albicans Candida* strains were incubated for 90 min, and nonadherent cells were removed by PBS washing. Subsequently, probiotic yeasts (10^8^/ml) were treated for 24 h at 37°C. These two experiments were considered to represent the initial stage of biofilm formation. Intermediate and mature biofilm treatments were conducted after 24 and 48 h of growth of non-*albicans Candida* biofilm, respectively (47). Similar treatments were performed for biofilms consisting of mixed *Candida* strains where C. albicans was used along with individual non-*albicans Candida* strains. The metabolic activity of biofilm was checked by MTT assays (48).

**(iii) *Ex vivo* adhesion assay in Caco-2 cell monolayers.** Adhesion to epithelial cells is a prerequisite for *Candida* to invade deeper tissues. To assess adhesion to live cells, we employed an *ex vivo* system using a Caco-2 cell monolayer (obtained from the National Centre for Cell Sciences [NCCS], India). The Caco-2 cells (5 × 10^4^ cells/ml) were seeded into a 96-well microtiter plate and incubated at 37°C in a 5% CO_2_ incubator for 20 days to produce a monolayer. The monolayer was further treated with probiotic isolates and non-*albicans Candida* strains under preinoculation, coinoculation, and postinoculation conditions, as explained above. Nonadherent cells were removed by washing with PBS (pH 7.4). Adhered cells were harvested using trypsin-EDTA (0.25% [wt/vol] trypsin and 0.02% [wt/vol] EDTA) treatment for 5 min at 37°C. Non-*albicans Candida* CFU levels were calculated on Candida chrome agar plates.

**(iv) *In vivo*Caenorhabditis elegans infection assay.** Eggs were harvested from six to eight worms reared on a Nematode growth media (NGM) agar plate containing E. coli OP50 and were incubated at 20°C for 3 days, and 40 to 50 harvested eggs were transferred to a fresh NGM plate that contained E. coli OP 50. The assay was conducted under coculture and postexposure conditions. For the coinoculation condition, 40 to 50 larval stage 3 (L3) and L4 worms were transferred into NGM plates containing probiotic yeasts and non-*albicans Candida* (10^6^ cells/20 μl as the test inoculum) and non-*albicans Candida* (10^6^ cells/20 μl as control) lawns, and the live and dead worms were counted each day using a dissection microscope. For the postinoculation condition, worms were exposed to non-*albicans Candida* species for 2 days (10^6^ cells/20 μl) and then transferred into a probiotic lawn (10^6^ cells/20 μl). Probiotic-treated non-*albicans Candida*-infected worms were compared with the control (non-*albicans Candida*-infected worms transferred into a OP50 lawn). Each day, dead and live worms were counted manually (49).

**(v) Non-*albicans Candida* colonization assay.** Averages of 5 to 6 worms grown under the coinoculation condition or the postinoculation condition or both were used in the study. Briefly, the worms were washed four times with PBS. The washed worms were then resuspended in 100 μl PBS buffer and crushed using a pellet pestle. Finally, the appropriate dilution of the sample was transferred into Candida chrome agar to differentiate non-*albicans Candida* strains from probiotic yeast isolates. Finally, levels of colonized non-*albicans Candida* cells were expressed in CFU per milliliter.

### Mechanism of probiotic effects on non-*albicans Candida* strains. (i) Two-chamber cell insert assay.

A dual-chamber cell insert apparatus with the chambers separated by a 0.4-μm-pore-size filter was used to evaluate the effect of the probiotic secretome on non-*albicans Candida* strain adhesion. Briefly, a total of 10^8^ cells/ml of S. cerevisiae, I. occidentalis, or S. boulardii was used to inoculate the upper chamber of the cell insert compartment, and 10^7^ cells/ml of non-*albicans Candida* strain were maintained in the lower chamber of the cell insert apparatus for 24 h with mild shaking. After incubation, the lower plates containing adhered non-*albicans Candida* strains were washed three times with phosphate-buffered saline (PBS; pH 7.4) to remove nonadherent non-*albicans Candida* cells followed by staining with the 0.5% crystal violet mentioned earlier. Finally, absorbance was measured at 590 nm to analyze the number of attached cells.

### Preparation of media for cell-free conditions.

Yeasts S. cerevisiae, I. occidentalis, and S. boulardii (10^7^ cells/ml) were inoculated into Synthetic Complete (SC) media and incubated at 30°C in 150 rpm for 3 days. Cell pellets were removed by centrifugation, and the supernatant was subjected to buffer neutralization and filter sterilization and was concentrated by lyophilization. The resulting medium, containing presumed bioactive molecules, was supplemented with SC media (at a ratio of 2:10) to replenish nutrients and was used in adhesion assays as described above.

### Statistical analysis.

Variations in treatments were compared using one-way analysis of variance (ANOVA) followed by *post hoc* analysis using Tukey’s *t* test at a significance level of *P* = <0.05. Results were expressed as means ± standard deviations (SD). Analyses were performed with GraphPad Prism 5 software (GraphPad Software Inc., San Diego, CA, USA). Kaplan-Meier statistical analysis tools were used for the C. elegans survival assay.
