# Supplementary figures and images for: Probiotic Yeasts Inhibit Virulence of Non-albicans Candida Species
Source: mBio. 2019 Oct 15;10(5):e02307-19. doi: 10.1128/mBio.02307-19 (PMC6794482; doi:10.1128/mBio.02307-19)

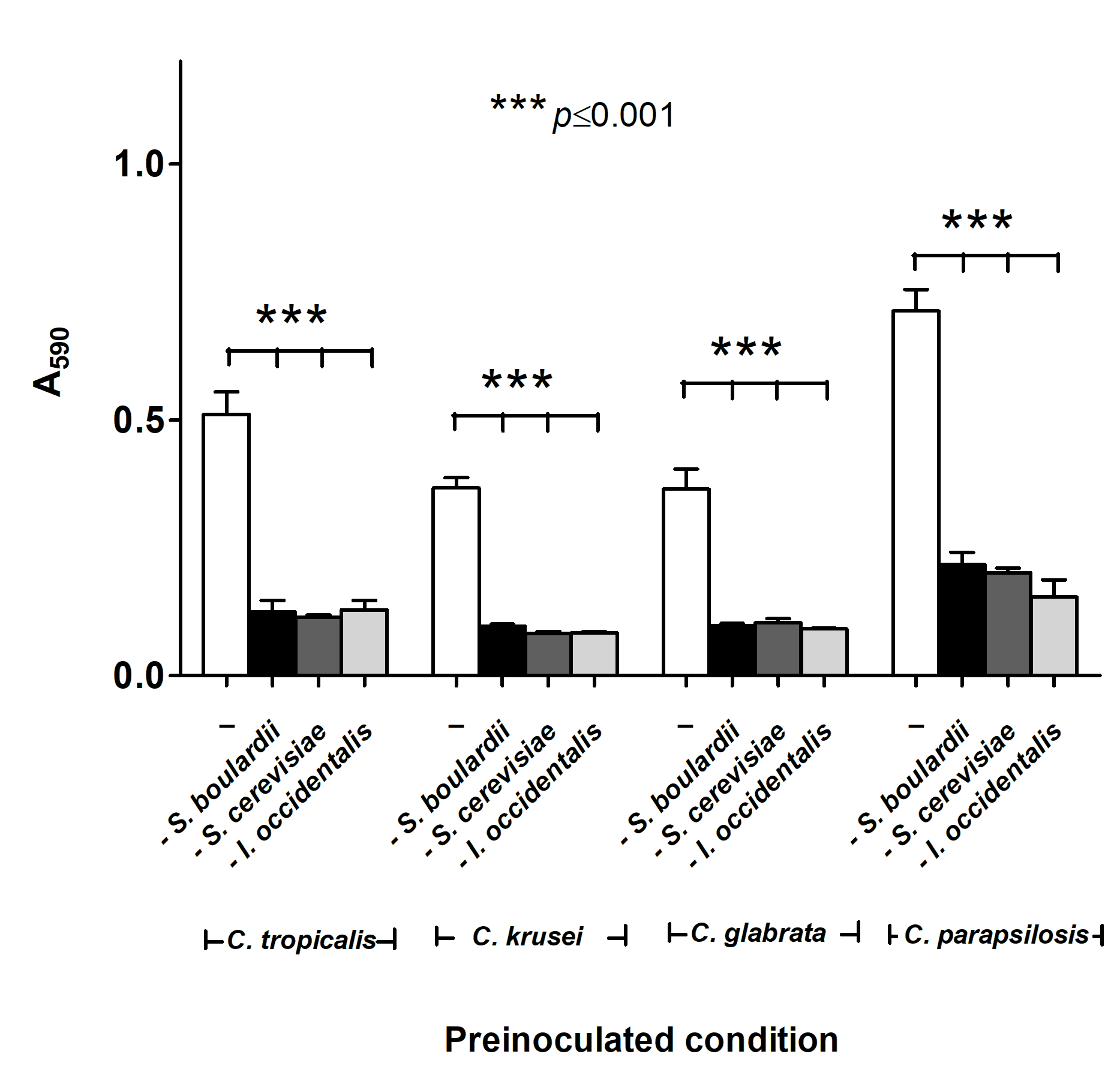

Supplement: FIG S1 [file mBio.02307-19-sf001.tif]

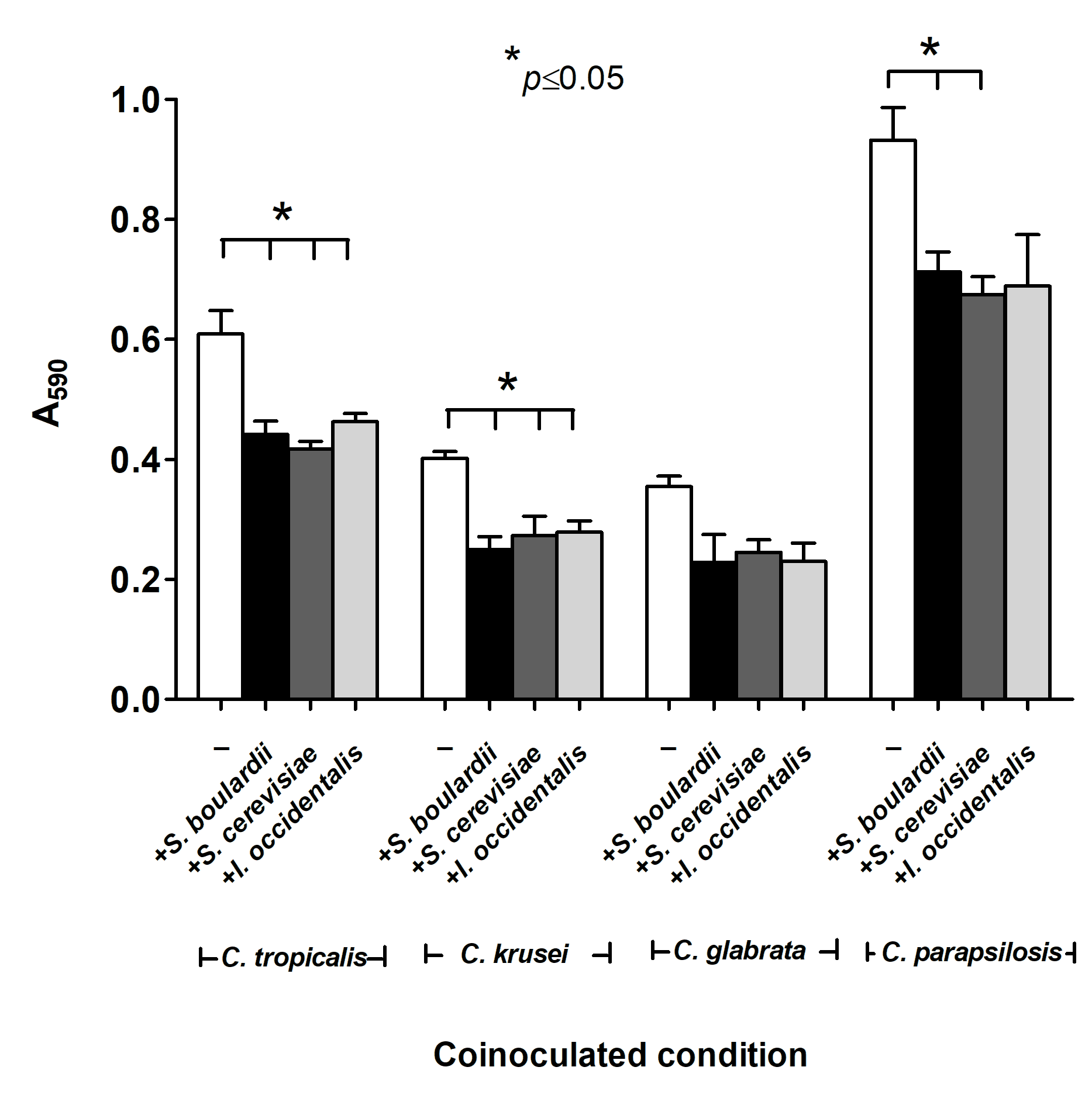

Supplement: FIG S2 [file mBio.02307-19-sf002.tif]

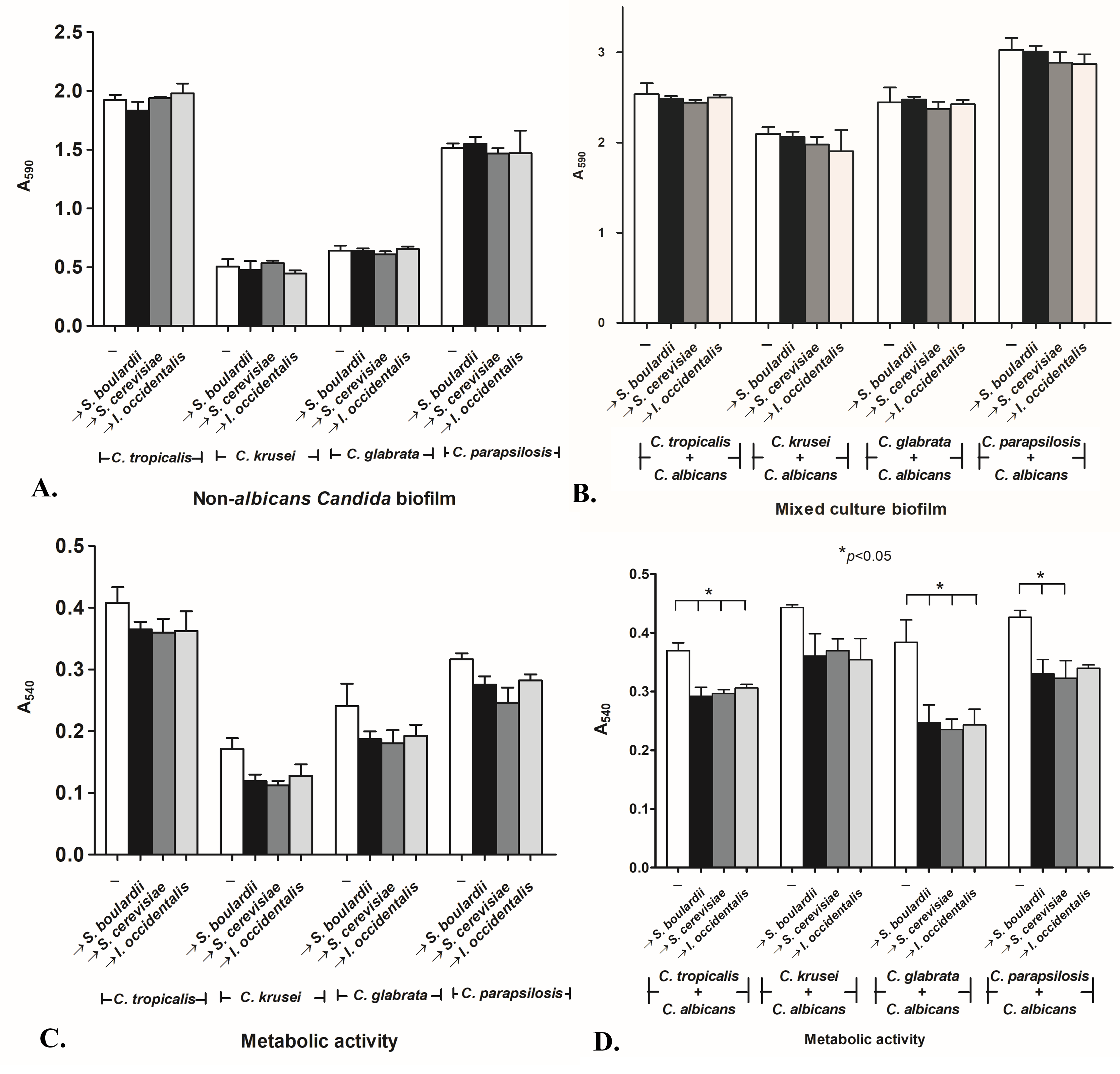

Supplement: FIG S3 [file mBio.02307-19-sf003.tif]

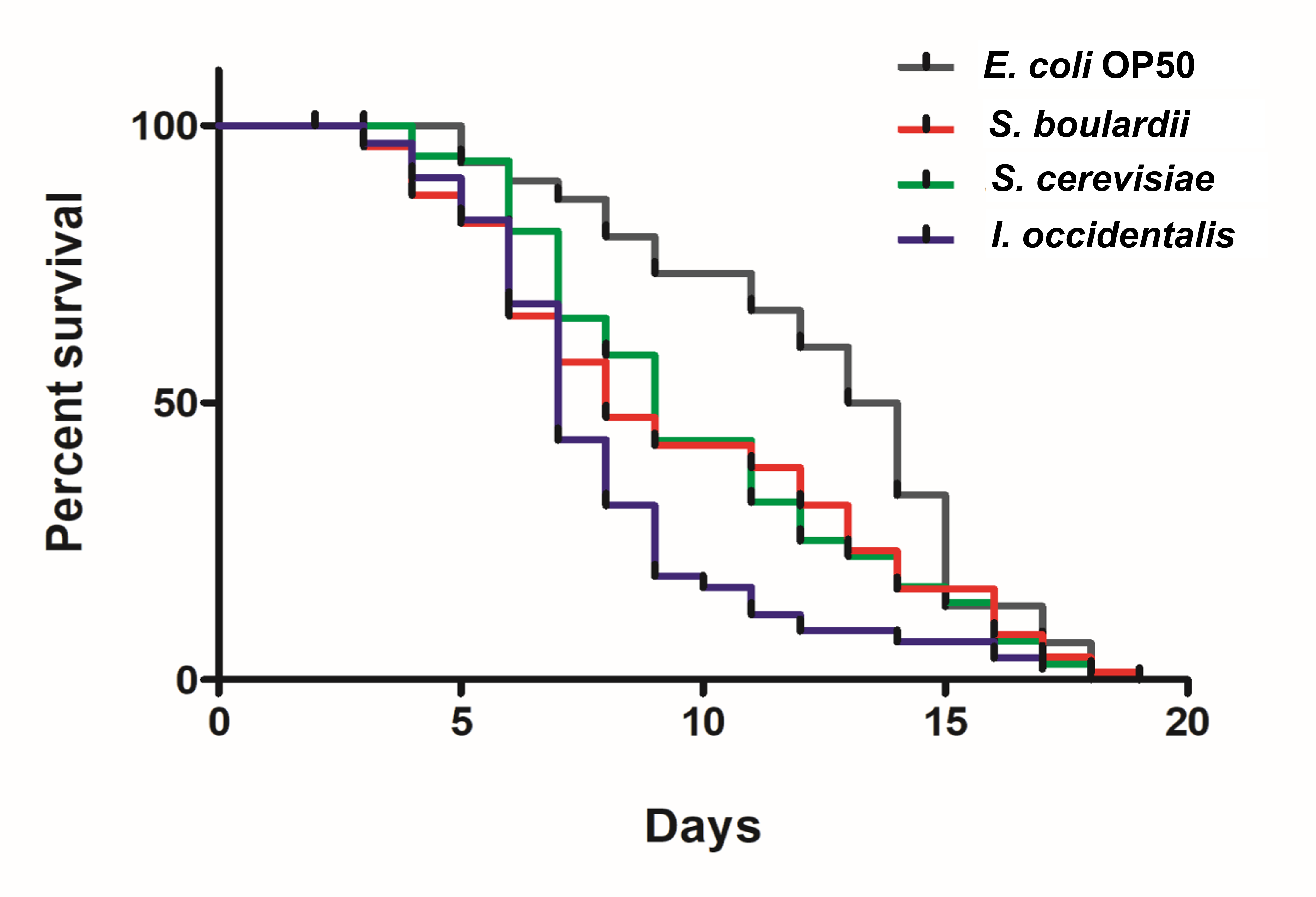

Supplement: FIG S4 [file mBio.02307-19-sf004.tif]

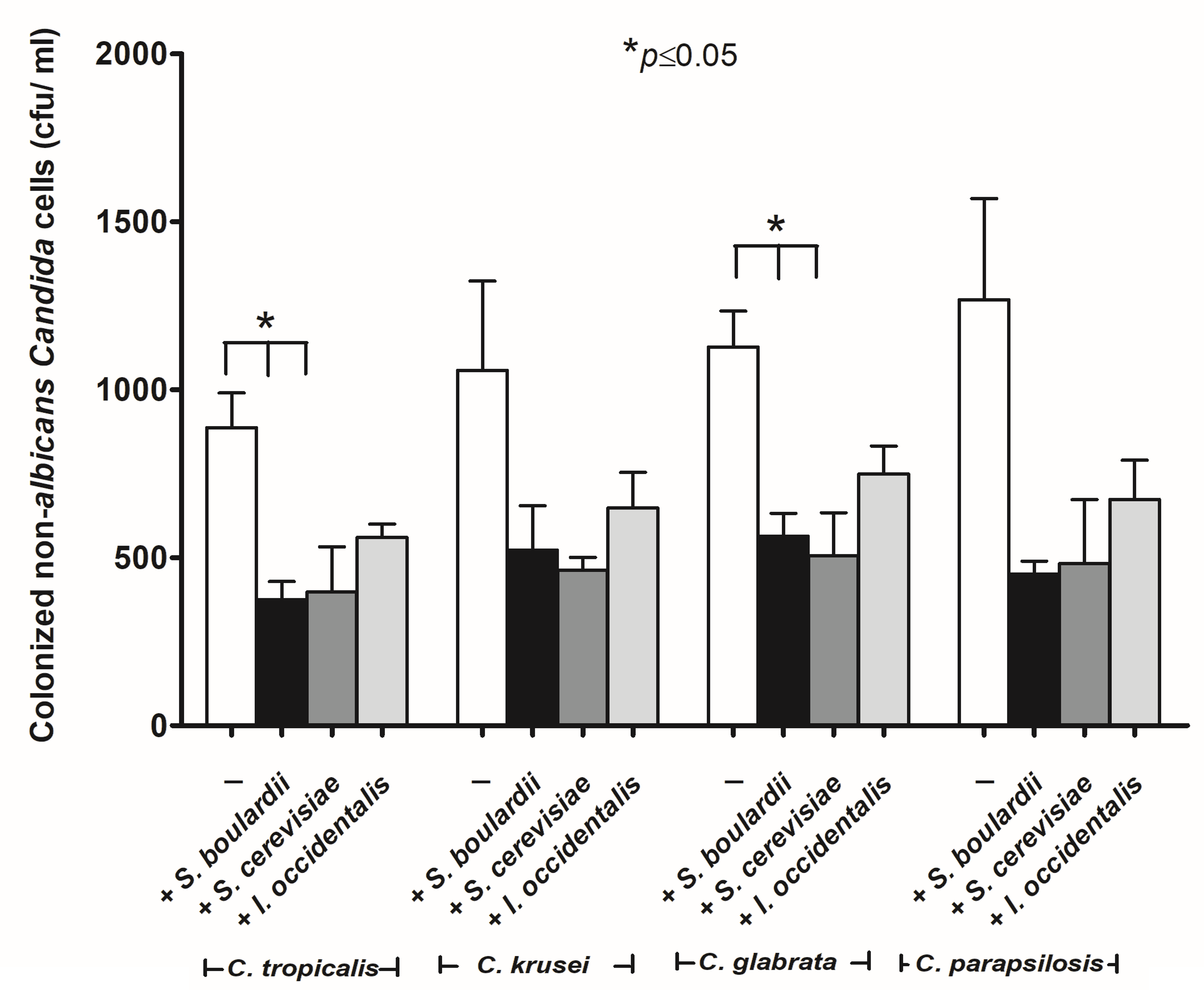

Supplement: FIG S5 [file mBio.02307-19-sf005.tif]
